# Antiviral Drugs for EBV

**DOI:** 10.3390/cancers10060197

**Published:** 2018-06-13

**Authors:** Joseph S. Pagano, Christopher B. Whitehurst, Graciela Andrei

**Affiliations:** 1 Department of Medicine, Lineberger Comprehensive Cancer Center, University of North Carolina at Chapel Hill, Chapel Hill, NC 27599, USA; 2 Department of Microbiology and Immunology, University of North Carolina at Chapel Hill, Chapel Hill, NC 27599, USA; cbwhiteh@med.unc.edu; 3 Department of Microbiology and Immunology, University of Leuven, BE-3000 Leuven, Belgium; Graciela.Andrei@rega.kuleuven.be

**Keywords:** Epstein–Barr virus, maribavir, antiviral, acyclovir, ganciclovir

## Abstract

Epstein–Barr virus (EBV) infects up to 95% of the adult human population, with primary infection typically occurring during childhood and usually asymptomatic. However, EBV can cause infectious mononucleosis in approximately 35–50% cases when infection occurs during adolescence and early adulthood. Epstein–Barr virus is also associated with several B-cell malignancies including Burkitt lymphoma, Hodgkin lymphoma, and post-transplant lymphoproliferative disease. A number of antiviral drugs have proven to be effective inhibitors of EBV replication, yet have resulted in limited success clinically, and none of them has been approved for treatment of EBV infections.

## 1. Introduction

Why is it that despite the profusion of drugs developed through the years that inhibit replication of the Epstein–Barr virus (EBV) their use clinically has been limited? The problem is illustrated by the commonest infection caused by EBV in Western countries: infectious mononucleosis (IM). First, its onset is insidious with nondescript sore throat, swollen lymph nodes, and splenic enlargement, along with lassitude that persists up to six months or longer while the virus has already been actively replicating for some time and shed from the oropharyngeal epithelial cells. Consequently, drugs given largely post-facto are too late. Infectious mononucleosis is essentially an immunologic condition triggered by EBV that is signaled by the atypical T-cell response in the blood. It therefore can be imagined that combined treatment with antiviral and immunosuppressive drugs might have an impact on infectious mononucleosis. However, in trials in which corticosteroid and antiviral drugs were administered together, the effects were marginal [1].

Acyclovir (ACV) was shown in 1982 to check replication of the virus with essentially no toxicity [2] because it selectively inhibited viral but not cellular replication. Its antiviral effective dose (ED_50_) was established as 0.3 μM with a cellular effect of 250 µM resulting in a highly favorable therapeutic index of 850 [3]. The antiviral effect of acyclovir is the result of ACV-triphosphate’s interaction with the EBV DNA polymerase with much higher affinity than for the cellular polymerase alpha. Acyclovir triphosphate is incorporated into the viral DNA where it forms a tight dead-end complex that stops irreversibly its chain elongation. Ganciclovir’s (DHPG) effect is even greater, but it is more toxic which may preclude its use in otherwise normal persons. It however can be valuable when used selectively.

## 2. EBV Latency and Antivirals

However, neither acyclovir nor other drugs have any effect on latent infection, which is dependent upon persistent EBV episomes, the circular form of EBV genome, not the encapsulated linear form [4]. The episome is replicated by the same mechanisms used by cells once every cell cycle, maintaining a stable number through successive generations. It is not itself oncogenic, but serves as the molecular basis of the latent state of EBV infection [5]. No inhibitors of EBV latent infection have materialized over the decades. Accordingly, despite prolonged suppression of viral replication, some latently infected cells will persist and will restore the population of the latent cells.

At the same time, non-toxic antiviral drugs are indispensable for treatment, and potentially prophylaxis, of infection in inborn and acquired immunodeficiency syndromes in which the latent genome has been reactivated. Reactivation in immunosuppressed individuals results in abundant viral replication that has the potential for genesis of B-cell lymphomas because of EBV’s ability to immortalize B-cells. In the immunocompetent, there is initially runaway B-cell proliferation, but it is normally checked by efficient T-cell responses.

## 3. Acyclovir and Infectious Mononucleosis

Acyclovir is a nucleoside analog as are penciclovir, ganciclovir, and their oral prodrugs. In some European countries along with brivudin (BVDU), they are approved for the therapy of herpes simplex virus 1 (HSV-1) and varicella-zoster virus (VZV) associated diseases. Although a number of other antiviral agents are effective inhibitors of herpesvirus replication, none of them have been approved by the FDA (Food & Drug Administration) or EMA (European Medicines Agency) for treatment of EBV infections [6].

In addition to its subtle onset, IM has a long incubation time (4–6 weeks), which results in late diagnosis in contrast to infections caused by HSV or VZV. Thus, the difficulty in the diagnosis of IM may be in part responsible for the lack of success in the development of a generally useful antiviral agent for EBV infection, except in immunodeficient states when there is active viral replication.

ACV does reduce EBV shedding in the oropharynx during IM, but is not accompanied by discernible clinical benefit. Diagnostically, IM is characterized by atypical T-cell lymphocytosis that results from the massive cell-mediated immune response against EBV-infected B-lymphocytes. Thus, it has been suggested that antivirals in combination with immunomodulatory drugs (such as corticosteroids, used empirically by physicians to treat IM) might be beneficial. However, in a multicenter, double-blind, placebo-controlled trial, prednisolone administered with ACV for treatment of IM inhibited oropharyngeal EBV replication without affecting the duration of clinical symptoms or development of EBV-specific cellular immunity [1,7].

The hepatitis associated with IM has been shown to be accompanied by a high viral burden [8,9], and accordingly specific antivirals could possibly alleviate symptoms of this common EBV-related complication, which is in any case, almost always benign and self-limiting.

## 4. Chronic Active EBV and Post-Transplant Lymphoproliferative Disease (PTLD)

Chronic active EBV infection is rare. It is characterized by a high EBV DNA load (10^3^–10^7^ copies/mL), indicative of active lytic viral replication [10]. It is unrelated to the fatigue experienced in IM that may last months to a year. Immunomodulatory agents (such as interferon-α and interleukin-2) have been used for treatment of chronic active EBV but with limited success.

Post-transplant lymphoproliferative disease is a potentially fatal complication following stem cell and solid organ transplantation. In EBV-naïve patients, the 30% to 50% who then seroconvert experience PTLD [11,12,13].

Most cases of early onset PTLD (occurring during the first year following transplantation) are associated with recent EBV infection. Late-onset lymphomas occurring after the first year are less likely to be associated with EBV [14]. The EBV immediately early (IE) proteins BZLF1 and BRLF1 contribute to cytokine IL-6’s secretion in lytically infected cells that may promote early lymphoproliferative disease [15]. IL-6 plays a crucial role in the maintenance of immune responses.

In 2002, Malouf and colleagues [12] reported a reduction in the incidence of PTLD in lung and heart-lung transplant recipients who received antiviral prophylaxis. They analyzed its incidence before 1996 and compared the impact of long-term antiviral prophylaxis on the development of PTLD in EBV-seronegative recipients between 1996 and 2000. In this study, none of the EBV-seronegative recipients receiving continuous antiviral prophylaxis developed PTLD, while in the historic group PTLD developed in 4.2% of the patients.

## 5. Ganciclovir and Valgancyclovir

The effects of ganciclovir (GCV) and valgancyclovir (VGCV) prophylaxis on EBV load was evaluated in a group of EBV-naïve pediatric renal transplant recipients who had received a graft from an EBV-positive donor, and therefore were at risk of primary infection with EBV [11]. Over the first post-transplant year, antiviral prophylaxis with VGCV or GCV resulted in a significantly decreased incidence of EBV primary infection: 9/20 (45%) in the prophylaxis group versus 8/8 (100%) in the non-prophylaxis group. There was a significantly lower EBV viral load depending on the type and intensity of the immunosuppressive therapy.

In a retrospective cohort study, the impact of antiviral prophylaxis (given to prevent human cytomegalovirus (HCMV) disease) in 73 EBV-seronegative adult kidney recipients on early and late PTLD onset was analyzed [16]. Thirty-seven patients received VACV or VGCV for 3–6 months and 36 received no-prophylaxis. Epstein–Barr virus PCR monitoring revealed that prophylaxis delayed primary infection at 100 days, but early PTLD incidence was not different between groups. However, regarding late events, EBV-related neoplasia incidence was significantly lower in treated patients among whom no cases were observed versus six cases reported in the no-prophylaxis group. Despite a weak level of evidence, this study suggests that antiviral prophylaxis could prevent late onset PTLD.

## 6. Omaciclovir

The carbocyclic nucleoside analog H2G, (*R*)-9-[4-hydroxy-2-(hydroxymethyl)butyl]guanine, omaciclovir, has potent activity against different herpesviruses, being particularly active against VZV [17]. Its mode of action is similar to that of ACV but omaciclovir has less selectivity as a substrate for thymidine kinase (TK). Resistance to H2G has been mapped in the VZV TK [18]. Unlike ACV, H2G is not an obligate chain terminator, although incorporation of its triphosphate form (H2G-TP) results in limited chain elongation. Also, H2G-TP has a longer intracellular half-life than ACV-TP.

## 7. Valomaciclovir

The L-valine ester of H2G, (i.e., MIV-606, (valomaciclovir, VALM)) is a prodrug of H2G with higher oral bioavailability than the parent compound. Phase I/II clinical trials with VALM (http://www.epiphanybio.com) include a randomized, placebo-controlled, double-blind trial for infectious mononucleosis [ClinicalTrials.gov Trial: NCT00575185]. Subjects over the age of 15 with acute IM were randomized to 21 days VALM 4 g/day (11 patients) or placebo (10 patients) and followed for six months. Valomaciclovir subjects had faster clinical improvement than placebo recipients did as documented by comparing the slopes of the plots of physical exam/symptoms scores from each visit during the treatment period. Severity of illness improved more quickly in the VALM group, but rates were not statistically significant. Valomaciclovir afforded a significant decrease in median EBV load in the oral compartment versus placebo mirroring ACV’s effect. The proportion of individuals who had at least a 2-log10 decrease in viral load in the oral compartment was also significantly greater in the treatment group than in the placebo group. This was the first study demonstrating the clinical benefit and the in vivo antiviral effect of VALM in acute IM.

## 8. Maribavir

Maribavir (MBV) is an investigational oral benzimidazole L-riboside with significant activity against both HCMV and EBV but not other herpesviruses [19,20]. Maribavir has more specific antiviral properties and fewer adverse side effects compared with currently approved anti-HCMV drugs. Unlike nucleoside and nucleotide analogs, MBV does not target the viral DNA polymerase. Its inhibitory effects are mainly produced through inhibition of the HCMV and EBV protein kinases (PK) [21,22]. This compound selectively inhibits the HCMV PK UL97 as point mutations in the UL97 gene confer MBV resistance in HCMV [23]. The first studies on GCV and MBV resistance showed that in vitro and in vivo UL97 mutations selected under GCV and MBV were distinct and conferred no cross-resistance [24,25], while there was partial cross-resistance between GCV and cyclopropavir (CPV). However, later investigations indicated that a single p-loop UL97 mutation can confer significant cross-resistance to the three drugs.

Maribavir has marked activity against EBV through a unique dual effect, inhibition of viral DNA replication and of virus transcription [6,26]. In contrast to HCMV, the activity of MBV against EBV could not be ascribed to direct inhibition of the EBV PK (BGLF4). In fact, MBV treatment was shown to inhibit the phosphorylation of the EBV DNA processivity factor (BMRF1) [20]. Unlike ACV that has little effect on EBV RNAs, MBV inhibits the expression of multiple RNAs. Furthermore, the inhibitory profile of MBV transcripts appeared to be similar to that produced by an EBV mutant in which PK expression and activity were knocked out [27]. The result suggested that EBV largely affects EBV transcript levels through inhibition of BGLF4, although MBV does not directly affect the EBV PK [28]. Considering that EBV BGLF4 has at least 20 viral targets, MBV may also affect downstream targets indirectly.

## 9. Cidofovir

Finally, there is some basis for using an antiviral drug in tumorous tissue. Cidofovir (HPMPC), a nucleoside analogue, has been used for treatment of papillomatosis in the hypopharynx and esophagus caused by human papillomavirus (HPV) [29]. This drug is not only known for its antiviral properties but also for its antiproliferative effects through a mechanism that remains unclear. In experiments with EBV-positive nasopharyngeal carcinoma (NPC) xenografts in nude mice, injection into the tumor tissue with cidofovir suppressed growth of the NPC tissue [30,31]. The ribonucleotide reductase (RR) inhibitors hydroxyurea and didox (3,4-dihydroxybenzohydroxamic acid) enhanced cidofovir-induced apoptosis in EBV-transformed epithelial cells and in EBV-positive nasopharyngeal carcinoma xenografts [31].

## 10. Thymidine Derivatives

L-dioxolane thymidine derivatives were described as a potent class of anti-EBV agents and their anti-herpesvirus activity was dependent on viral TK. The HDVD analog (1-[(2*S*,4*S*-2-(hydroxymethyl)-1,3-dioxolan-4-yl]5-vinylpyrimidine-2,4(1H,3H)-dione) and its oral prodrug demonstrated also efficacy in a mouse model of MHV-68 (a surrogate of EBV), reducing viral replication in the lungs [32].

Two novel thiothymidine derivatives, KAY-2-41 and KAH-39-149, were particularly active against EBV in addition to their inhibitory effects against HSV and VZV [33]. Their antiviral activity proved to be dependent on phosphorylation by viral TK and by cytosolic TK1. The efficacy of both thionucleosides in mice against MHV-68 acute infection was mostly promising for KAH-39-149, which showed similar in vivo potency as cidofovir.

## 11. Conclusions

Approximately 95% of the world’s population is infected with EBV, but most do not present disease. However, for those that do develop EBV-related illness there remains no directed small molecule therapy as antivirals used in clinical trials have been largely ineffective. Past antiviral attempts have primarily targeted viral replication and further research into new avenues of study, including targeting non-replication proteins, may be necessary to develop effective therapy for EBV infections. EBV-triggered disease can cause debilitating illness and death and antiviral therapy targeting EBV for all related indications remains a major unmet medical need.
